# A Low-Cost Method for Phenotyping Wilting and Recovery of Wheat Leaves under Heat Stress Using Semi-Automated Image Analysis

**DOI:** 10.3390/plants9060718

**Published:** 2020-06-05

**Authors:** Agata Rascio, Giuditta De Santis, Giuseppe Sorrentino

**Affiliations:** 1 Council for Agricultural Research and Economics-Research Centre for Cereal and Industrial Crops, S.S. 673 Km 25,200, 71122 Foggia, Italy; giuditta.desantis@crea.gov.it; 2 ISAFOM, National Research Council, via Patacca 85, Ercolano (NA), 80056 Naples, Italy; giuseppe.sorrentino@isafom.cnr.it; 3 Institute for Sustainable Plant Protection, Via Università 130, 80055 Portici (NA), Italy

**Keywords:** wilting, heat stress, automated imaging, wheat, kinematic analysis

## Abstract

Leaf wilting is the most common symptom of dehydration stress. Methods to analyze this phenomenon are particularly relevant to evaluate crop agronomic performance, to genetically dissect out the wilting process, and for functional analysis of genetically modified plants. In this study, a low-cost, semi-automated method to quantify leaf folding of wilting plants is described that can replace visual analysis. Standardized heat-stress conditions were applied with a thermostatic drier, on plantlets or excised leaves from three wheat genotypes (Trinakria, Cappelli, and a Water-mutant of Trinakria). The best time–temperature binomial to record both the leaf wilting and recovery phases was identified using a free time-lapse application, by a smartphone camera. The quantitative description of the wilting phenomenon was obtained through the Kinovea software, which automatically tracked the leaf angle changes over time, computed various kinematic data (angular velocity, centripetal acceleration, total degrees of displacement) and constructed the graphs. The possibility of applying standardized heat-stress conditions and quantitatively describe the leaf folding kinematics means that this instrumentation and its use represents a very low cost tool for objective phenotyping of the degree of the heat-stress tolerance of wheat and of morphologically similar species.

## 1. Introduction

Wheat is the most cultivated cereal of the EU crops [1], where it represents up to ~50% of total production. However, in Mediterranean areas its vulnerability to climate changes and droughting is increasing [2]. Through phenotyping, which aims to monitor the quality, photosynthesis, development, architecture, growth or biomass productivity of single plants or plant stands [3], efforts have been made to select more suitable genotypes for the Mediterranean environment, although the specific phenotyping requirements for wheat have not been fully identified to date [4].

The most visible effect of dehydration-stress-induced wilting is leaf folding. This can occur as a consequence of accelerated water loss through the stomata, or insufficient uptake or water flux through the plant. By changing the leaf inclination, the plant can reduce the incident radiation, and hence the leaf surface temperature, the leaf transpiration, but also photosynthetic activity, and shading of basal leaves. For this reason, studies of wilting processes on whole plants or excised leaves have been widely carried out for the development of more abiotic-stress-tolerant crops, at the field scale and indoors [5,6,7,8,9]. The controlled environment has the advantage of allowing better control and reproducibility of experimental conditions, because the wilting symptoms can change with stress type and species, and also leaf age. As an example, wheat leaves under dehydration stress generally bend down [10], but flag and penultimate leaves longitudinally roll and remain rigid [11].

Differential wilting has been traditionally estimated by visual scoring and quantified by arbitrary scales [5,6,7], but this method is affected by the subjective interference of the analyst. Innovative, phenotyping technologies are now available like: RGB visible, infrared, fluorescence, or thermal imaging [12,13], which were originally established in remote sensing and in precision agriculture. These technologies are non-invasive and generally less labor intensive. They also offer the advantages of a more organic physiological analysis, as not only leaf morphology can be examined, but also leaf photosynthetic activity, stomatal behavior, or water status [14]. On the other hand, their costs depend on equipment, on the number and complexity of operations associated with the observation of a given set of phenotypic traits [15,16]. 

Dynamic imaging of single plants requires detailed views obtained using expensive tools, like dedicated digital time-lapse cameras, along with the support hardware, byte-wide memory to store data, and/or species-specific equipment [16,17]. For example, a laser scanner driven by two stepper motors positioned on an aluminum frame [18] was used for 3D wilting imaging of the wide zucchini leaves, and a front view from a set of cameras was used for rhythm tracking of leaf movements of six different plant species [19], with fixed focus and a minimum per plant pixel count of 10,000 (100 × 100 pixels). Finally, large image series that can be acquired during video recording have to be post-processed through commercial software or algorithms developed ad hoc [17,19]. The accurate capturing of the wheat leaf tips is considered to be the “single most crucial, open problem, despite progress achieved in imaging methods for plant phenotyping” [20,21].

With the aim to develop a low-cost method to quantitatively, and hence objectively, analyze leaf movements during and after strong heat shock, this study describes such a user-friendly protocol for quantitative comparisons of wheat leaf folding. The system developed consists of a thermostatic chamber to impose stress, a smartphone equipped with an ‘App’ (Application) for time-lapse recording, together with the free Kinovea software, a video player ideated for movements in sport analysis. Heat stress was simulated on plantlets or excised leaves of three wheat (*Triticum turgidum* L. ssp. *durum*) genotypes: Trinakria, Cappelli, and a Water-Mutant of Trinakria. By recording the changes in the leaf angle over time, the protocol was effective for the differential determination of several kinematic parameters, including: onset time of leaf folding (under stress) and unfolding (during recovery); maximum leaf-angle change; rate of folding under stress and unfolding during recovery.

## 2. Results

### 2.1. Wilting of Plantlets on Soil

**Figure 1:**
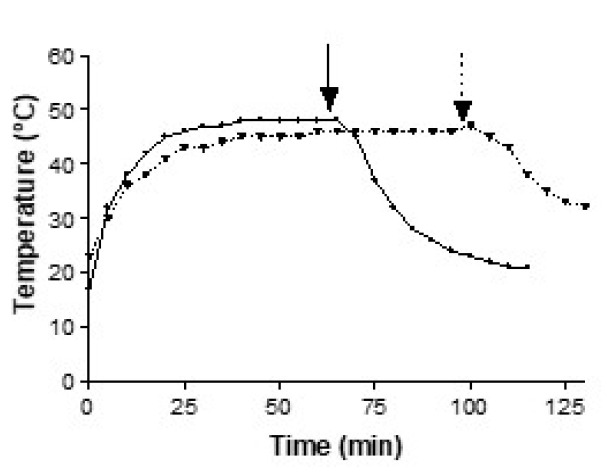
Temperature changes over time in the plant dehydration chamber measured in two different experiments with short (solid line) and prolonged (dotted line) heat shocks. The arrows indicate when the dryer was switched off, for the plant recovery phase.

Starting from room temperature (~20 °C) and setting the thermostat to 60 °C, the temperature near the plants reached the maximum (~48 °C) in 20 min (Figure 1). Once the switch was off, the temperature returned to 20 °C in ~50 min.

**Figure 2:**
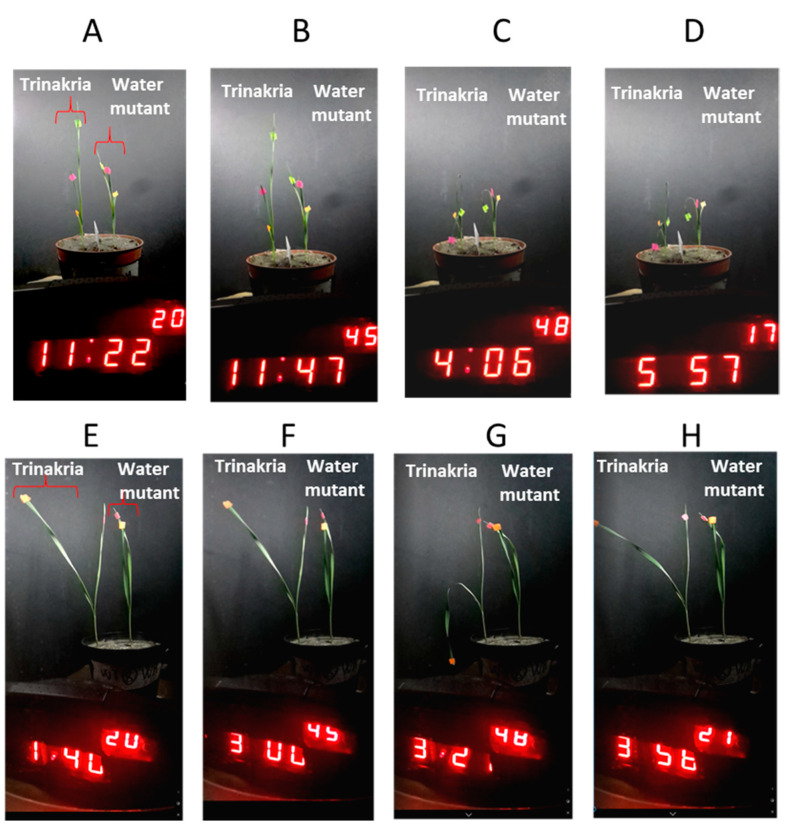
Wilting of Trinakria and Water-Mutant plants grown in soil and exposed to from 45 to 48 °C for ~5 h (**B**–**D**) or ~2 h (**F**–**G**). Images show plants before stress imposition (**A**,**E**), during the temperature increase (**B**,**F**), at the end of the stress cycle (**C**,**G**), and sometime after the stress relief (**D**–**H**).

To define the best time–temperature binomial that allowed the recording of both the wilting and recovery of plants grown on soil, the performance of Trinakria and its Water-Mutant were analyzed under the same stress intensity (48 °C) applied for long (Figure 2A–D) or short (Figure 2E–H) times. In the first experiment, both of the genotypes were irreversibly wilted (Figure 2C), and hence unfolding of the leaves was not seen, even up to 2 h after the dryer was switched off (Figure 2D). When a shorter stress duration (~2 h) was used, both leaf folding (Figure 2G) and unfolding (Figure 2H) were recorded for the second leaf of Trinakria.

**Figure 3:**
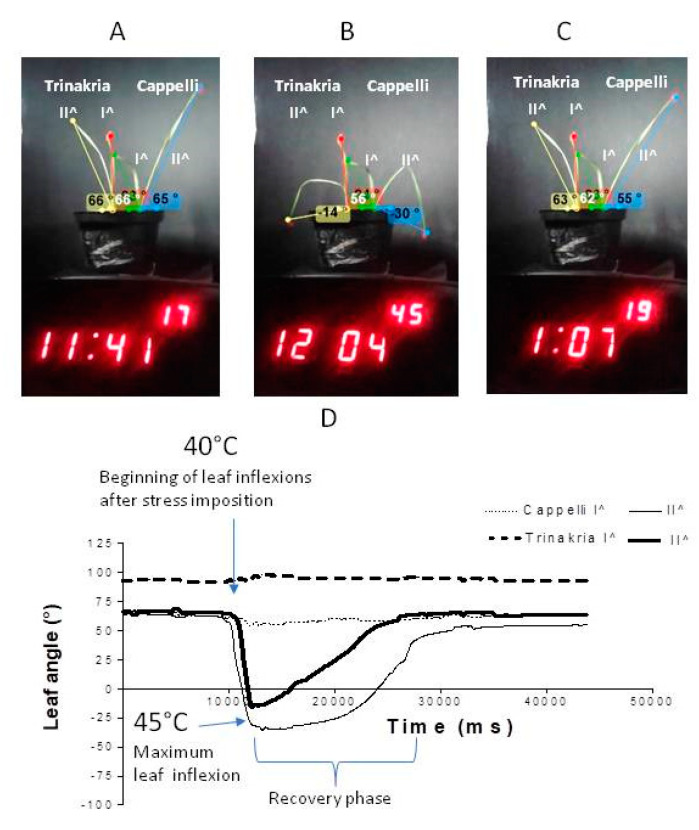
Wilting and recovery of Trinakria and Cappelli plants grown in soil. (**A**–**C**) Leaves with tracked angles extracted by Kinovea software analysis of the time-lapse video, recorded during stress imposition. Plants are shown before the heat shock (**A**), under heat stress (**B**), and at the end of the recovery phase (**C**). (**D**) Quantitative representation of the leaf angle changes as a function of movie time, from the Kinovea software analysis.

The computerized elaboration of all of the video frames showed that the vibrations of the plants due to the thermostatic chamber fan and the slow airstream speed did not prevent the Kinovea software from extracting and tracking the leaf angles (Figure 3; Supplemental Video S1). After saving all of the data, the graphical representation of the kinematic data allowed the quantitative depiction of the wilting process to be measured.

Table 1 gives the mean kinematic parameters concerning the four replicated wilting experiments on the Trinakria and Water-Mutant plants. The results of the *t* test confirmed that the two genotypes began to fold their leaves at the same time from the beginning of the temperature increase. In contrast, the absolute value of maximum inflexion angle of the leaves and the folding velocity were significatively higher for the wild-type vs. Water-Mutant, which also showed slower unfolding velocity, and failed to recover ~85% of the starting leaf angle.

To define the repeatability of the folding mode for Trinakria and its Water-Mutant, the differential wilting was also examined, comparing the Trinakria and Cappelli plants (Figure 3A–C). As for the short stress shown in Figure 2E–H, both of the first leaves were largely unaffected, while the second leaves of Cappelli and Trinakria bent downward with progressing dehydration stress (Figure 3B). After switching off the thermostat, all of the leaves recovered their turgidity. The comparison of the results of Figure 2G and Figure 3B shows that when Trinakria was exposed to heat stress with Cappelli, its leaf folding occurred at a lower temperature compared to when Trinakria was exposed to heat stress with its Water-Mutant, with the following parameters: leaf maximum angle change (Cappelli, ~97°; Trinakria, 78°), rate of folding under stress (Cappelli, 0.028°/ms; Trinakria, 0.026°/ms) and unfolding rate (Cappelli, 0.0047°/ms; Trinakria, 0.055°/ms).

**Figure 4:**
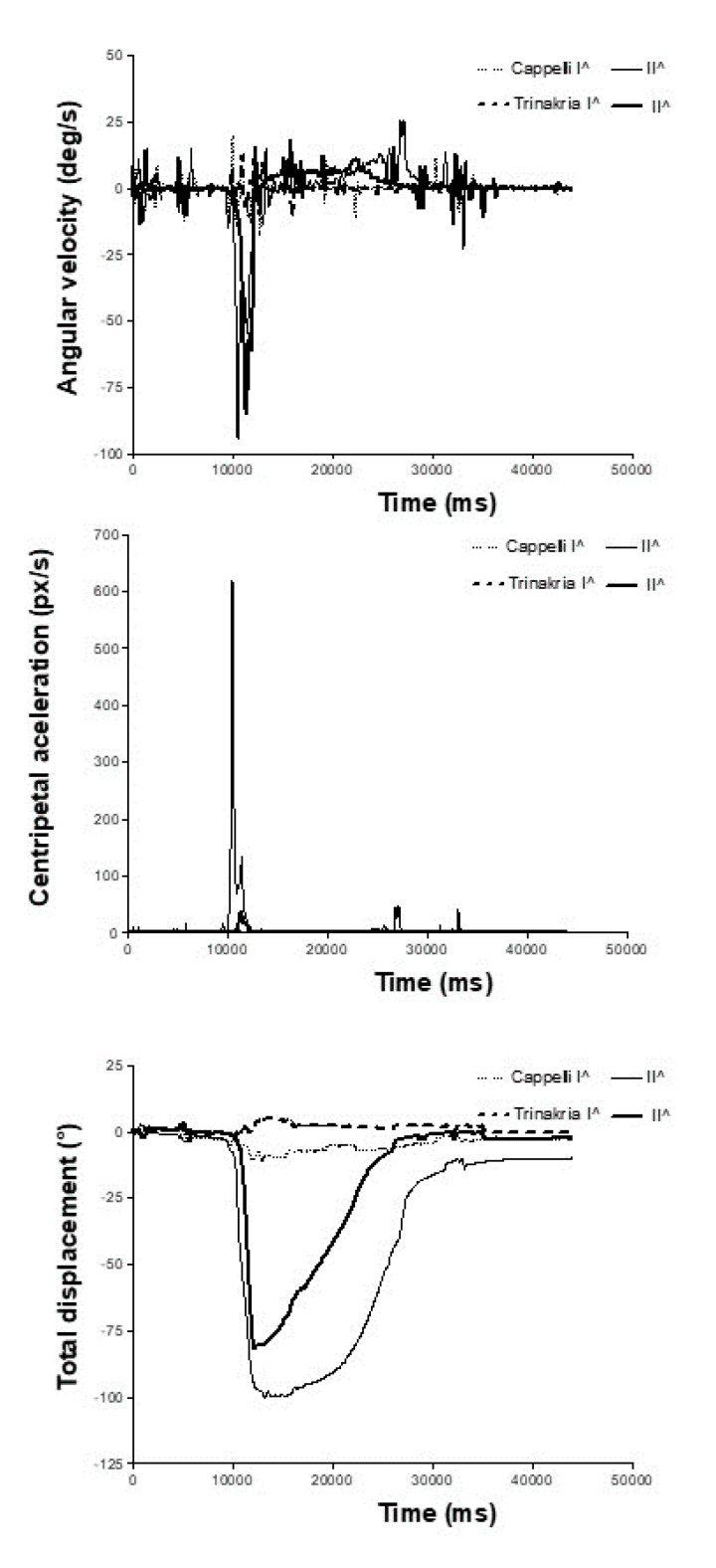
Further elaboration for wilting and recovery of Trinakria and Cappelli plants grown in soil, produced by the Kinovea software: leaf angular velocity (**top**), centripetal acceleration (**middle**), and total degrees of displacement (**bottom**) as a function of movie duration.

Figure 4 shows some further data elaboration produced by the Kinovea software in addition to those shown in Figure 3, as: leaf angular velocity, centripetal acceleration, and total degrees of displacement. These data above all highlight the large differences between the leaf types for the folding velocity, both under the stress conditions and for the recovery.

### 2.2. Wilting of Plantlets without Soil

**Figure 5:**
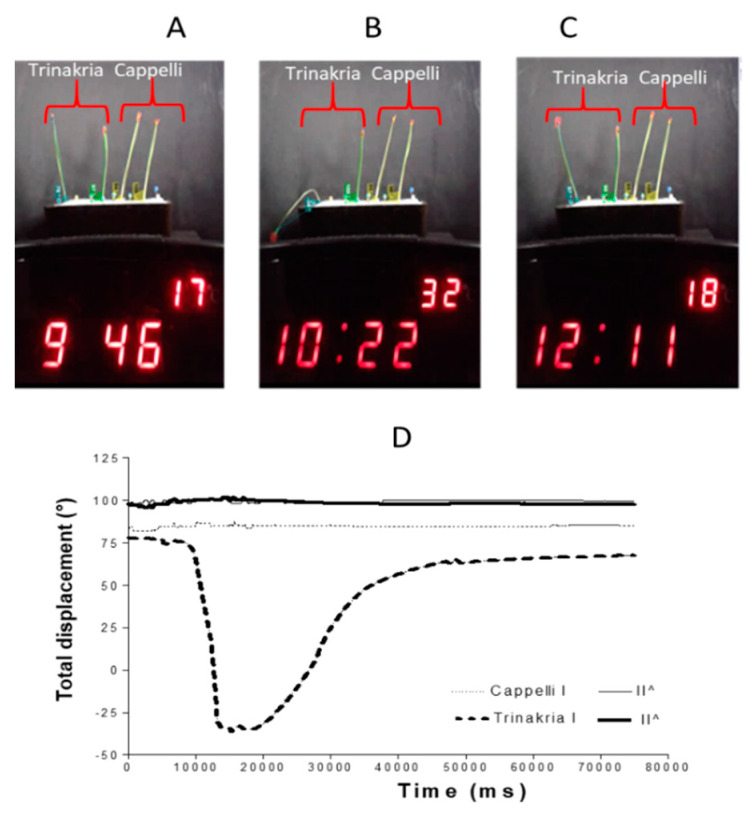
Wilting of whole plantlets germinated in paper rolls. Representative experiment with two Trinakria leaves and two Cappelli leaves, shown before the heat shock (**A**), at the maximum stress level at 32 °C (**B**), and following the recovery after stress relief (**C**). (**D**) Quantitative representation of the leaf angle changes as a function of movie time, from the Kinovea software analysis duration.

The leaf folding under heat shock and recovery for two Trinakria plantlets (I, II) and two Cappelli plantlets (I, II) germinated in paper rolls are shown in Figure 5A–C. As the roots were not immersed in water, only a moderate temperature increase (32 °C) induced earlier and larger leaf angle changes for Trinakria, while the subsequent lowering of the temperature and water addition allowed the full turgor recovery of the Trinakria leaves. As indicated above, all four of the leaf angles were correctly traced, including for the recovery phase. The kinematic data well reflected the differential leaf-folding dynamics (Figure 5D).

### 2.3. Single Leaf Wilting

**Figure 6:**
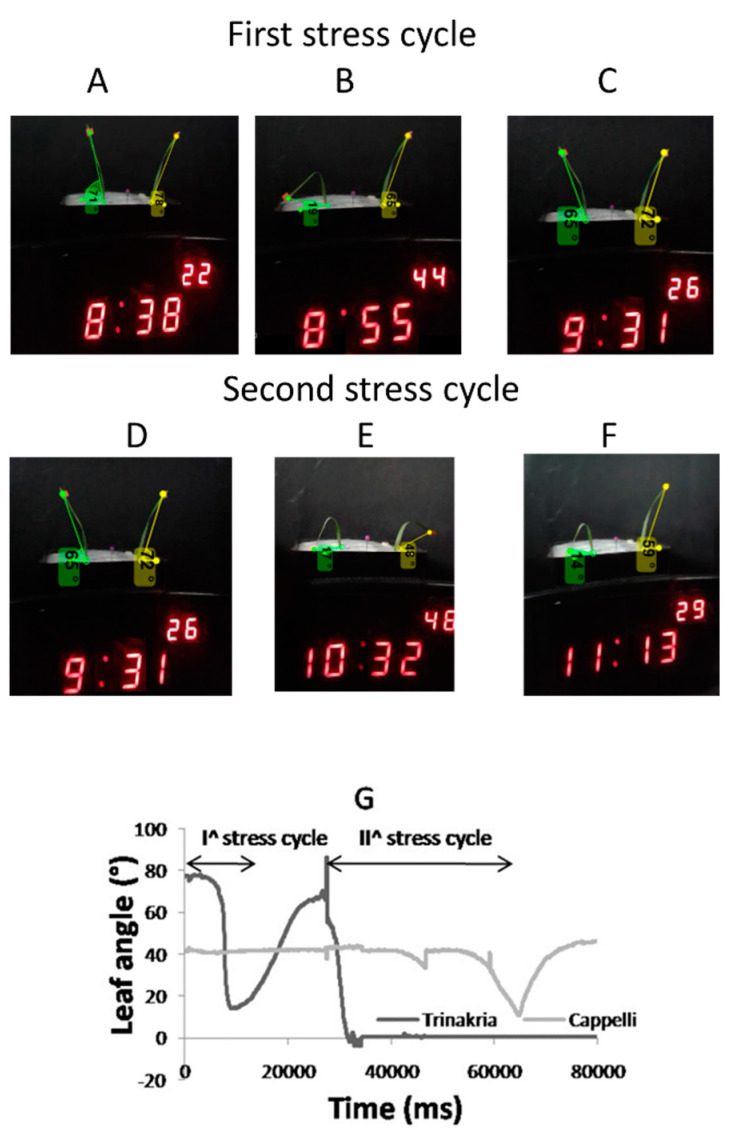
Wilting of single leaves excised from Trinakria (left in each) and Cappelli (right). Second (basal) leaves were collected from plantlets and subjected to two stress cycles, of 22 min (I) and 60 min (II). These show the leaves before the heat shock (**A**,**D**), at the maximum stress levels (**B**,**E**), which occurred at 44 °C for the first cycle (**B**) and 48 °C for the second cycle (**E**), and following the leaves after the stress relief (**C**,**F**). (**G**) Quantitative representation of the leaf angle changes as a function of movie time duration, as analyzed with the Kinovea software.

Figure 6 shows images from a wilting experiment using single excised second leaves (Figure 6A–F) from plantlets of Trinakria (left) and Cappelli (right). Two stress cycles were applied here. The first cycle (Figure 6A–C) only had visible effects on the leaf of Trinakria (Figure 6B) and these effects were reversible (Figure 6C). The second stress cycle (Figure 6D–F) lasted 1 h. Both of the leaves folded, but only the wilting with Trinakria was not reversible. The representation of the kinematic data in Figure 6G, as elaborated from Kinovea, thus shows the Trinakria curve with two inflection phases, where the second was irreversible. Instead, the Cappelli leaf only flexed during the second stress cycle and then fully recovered after heat-stress removal.

**Figure 7:**
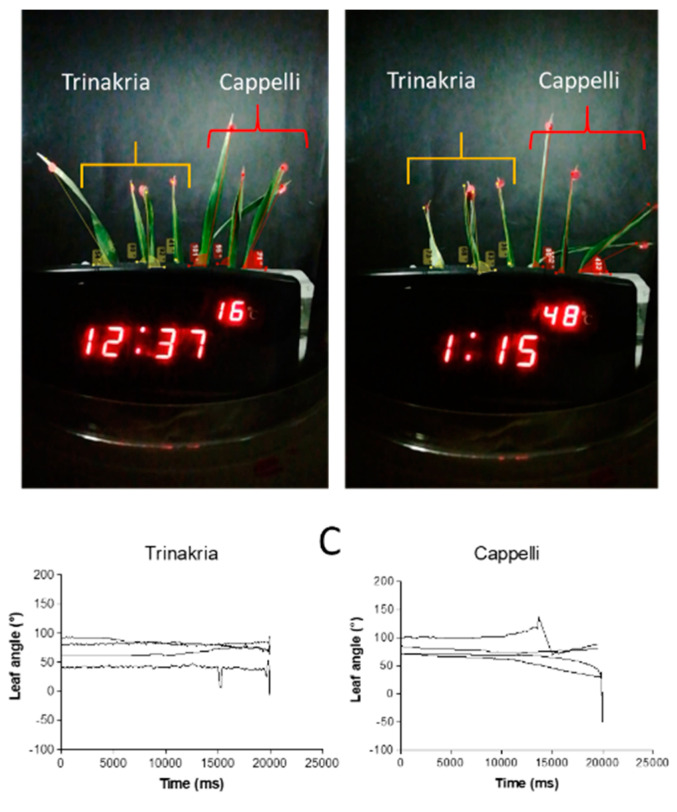
Genotypic comparison for the wilting of flag leaves excised at heading, from plants grown in the field. Images of tracked angles on leaves of four leaves of Trinakria and four leaves of Cappelli are shown, before (**A**) and after (**B**) heat-stress imposition. (**C**) Quantitative representation of the mean (*n* = 4) leaf angle changes as a function of movie time length, as elaborated by the Kinovea software.

The effects of a stress cycle applied to full expanded flag leaves excised from plants of Trinakria and Cappelli grown in the field are shown in Figure 7A,B and Supplemental Video S2. The leaves were collected from old plants and had more complex movements than leaflets, because both rolling and flexing were seen. The kinematics of whole process are summarized in Figure 7C, where the leaf movements appeared to be faster and larger for Cappelli than Trinakria.

## 3. Discussion

Leaf folding has long been used as an indicator of wilting, and hence of unfavorable water status in herbaceous plants [22]. The appearance of wilting symptoms can be a consequence of complex physiological and metabolic plant reactions to stress [23], and biological differences exist among cereal species and varieties [24] that might affect their permanent wilting points.

The development of low-cost methods to establish whether the same type of stress is non-lethal or lethal are relevant for breeders to functionally analyze genetically modified herbaceous plants, breeding lines, and mapping populations, for example. Innovative automated platforms for indoor and in-field plant phenotyping at different levels of biological organization [13] have been developed, although their use has often been impaired by the high costs of the required infrastructure and the limited availability of skilled personnel [5]. Low-cost instrumentation is available to simulate elevated night temperatures [25]. Other methods can analyze the wilting of large leaves or can capture plane images of plant stands under stress conditions, but compared to the present method, they require more facilities, such as an IR camera, a 3D scanner and an aluminum frame and/or a four-step pipeline for image processing [14,18,26]. Some studies have specifically solved the problem of wheat leaf segmentation [21] or constructed sophisticated models of stress-induced leaf deformation using computer graphics [27,28], to provide the potential for their use for physiological studies of dehydration effects. None of the above-cited studies, and indeed no others, to the best of our knowledge, can simultaneously solve the multiple critical stages of a differential wilting analysis of thin leaves using low-cost instrumentation like the present method. As described here, this is appropriate for imaging the wilting of wheat leaves, and also has potential for other species with similar morphologies, like fescue, rice, barley, and others. Indeed, due to the prevalence of length over the other dimensions, when the narrow and thin leaves of wheat fold down, their curvature can be faithfully reproduced in 2D imaging, using a smartphone camera with a resolution of 1920 × 1080 pixels. Using the low-cost thermostatic chamber, controlled and reproducible heat stress can be simulated. The treatment can be applied to whole plants, and also to excised leaves, with the further advantage that the genotypic intrinsic properties of the leaves can be compared without other interference, in terms of plant architecture, development, or functional activities of the roots. Using the smartphone memory, which also allows one to save and simply transfer the video file to a computer through a USB cable, the automatic recording of the wilting phenomenon and adequate data storing capacity is achieved. The leaf-tip movement tracking and saving by the Kinovea software avoids intensive image post-processing and kinematic data elaboration, which is necessary to obtain a quantitative measurement of the differential wilting trends. Finally, a very important feature of this protocol is that the whole equipment can be used by unskilled personnel. However, a limiting aspect of our prototype is the dimension of the dehydration chamber, which allows simultaneous comparisons of two plants, or four seedlings, or eight adult leaves, at a time. Due to the very low cost of the experimental equipment, a set of larger dehydration chambers can be used simultaneously to speed up the analysis time and to obtain the representative number of plants for a complete physiological analysis.

Although the main aim of this study was to test the effectiveness of the method to track and to quantitatively measure the differential wilting, some evidence emerged that leaves of different genotypes fold in the same way, with basal leaves moving down more rapidly than apical leaves. Depending on stress intensity, leaf age, and genotype, the following might occur: no leaf flection, more or less rapid leaf angle changes, and reversible and irreversible leaf folding. The comparison of the two genotypes Trinakria and its Water-Mutant that differ due to their tissue affinity for water [29] showed a reproducible tendency of the Water-Mutant to maintain the more vertical position of the leaves under heat stress and to recover the vertical position after the stress removal; conversely, the time when external signs of wilting appeared was similar for the two genotypes. These observations suggest to us the need to determine whether these genotypes differ more in terms of the plant reaction to stress, than to stress perception. Analogously, it should be necessary to determine whether the wilting trend can change with genotypes that are sown together.

Further studies are planned to verify these observations and to cross-validate the effectiveness of this low-cost method to define the status of the plants under stress, by simultaneous measurements of other physiological parameters (e.g., photosynthetic activity, water status) using contactless methods [14,20].

In conclusion, considering the vastness and completeness of quantitative data that can be obtained by this simple cost-effective system and its easy-to-use protocol, it might be a powerful tool for the accurate and objective phenotyping of wheat and species with similar morphologies for differential wilting, a process that is essential for the adaptation of crops to dehydration stress.

## 4. Materials and Methods

Two commercial durum wheat (*Triticum turgidum* L. ssp. *durum*) varieties, Trinakria and Cappelli, were used because they have very different genetic backgrounds and are traditionally cultivated in drought-prone areas of southern Italy. For the preliminary evaluation of functional effects in terms of the wilting tendency of a mutated phenotype, a Water-Mutant of Trinakria [29] was also analyzed. This Water-Mutant has a very negative hydration enthalpy of bound water, while the water fraction is essential for the structural integrity of biomolecules [30] that might also exert passive control of the osmotically active volume of the cell [31]. All of the genotypes were sown indoors (as described below) and in 10-m^2^ large plots of the experimental fields of CREA-CI, of Foggia (Italy), as previously reported [32].

### 4.1. Seed Germination

Surface-sterilized seeds (2% sodium hypochlorite, 5 min) of each genotype were washed several times with boiled distilled water and then germinated using the paper roll method [33]. Ten seeds were aligned on the upper side of a rectangular (20 × 50 cm) piece of filter paper (Whatman number 1), which was rolled and tightly wrapped with a plastic film, to avoid seed movements and dehydration. Then, 20 mL distilled water was added to the upper side of the paper rolls. These were positioned vertically in a beaker containing 1-cm depth deionized water, which was put in a drier and then in a growth chamber, at 20 °C/18 °C. A 14/10 h light/dark period was obtained with LED lamps. The radiation measurement at the pot surface was 60 µmol m^−2^ s^−1^ (400–700 PAR).

**Figure 8:**
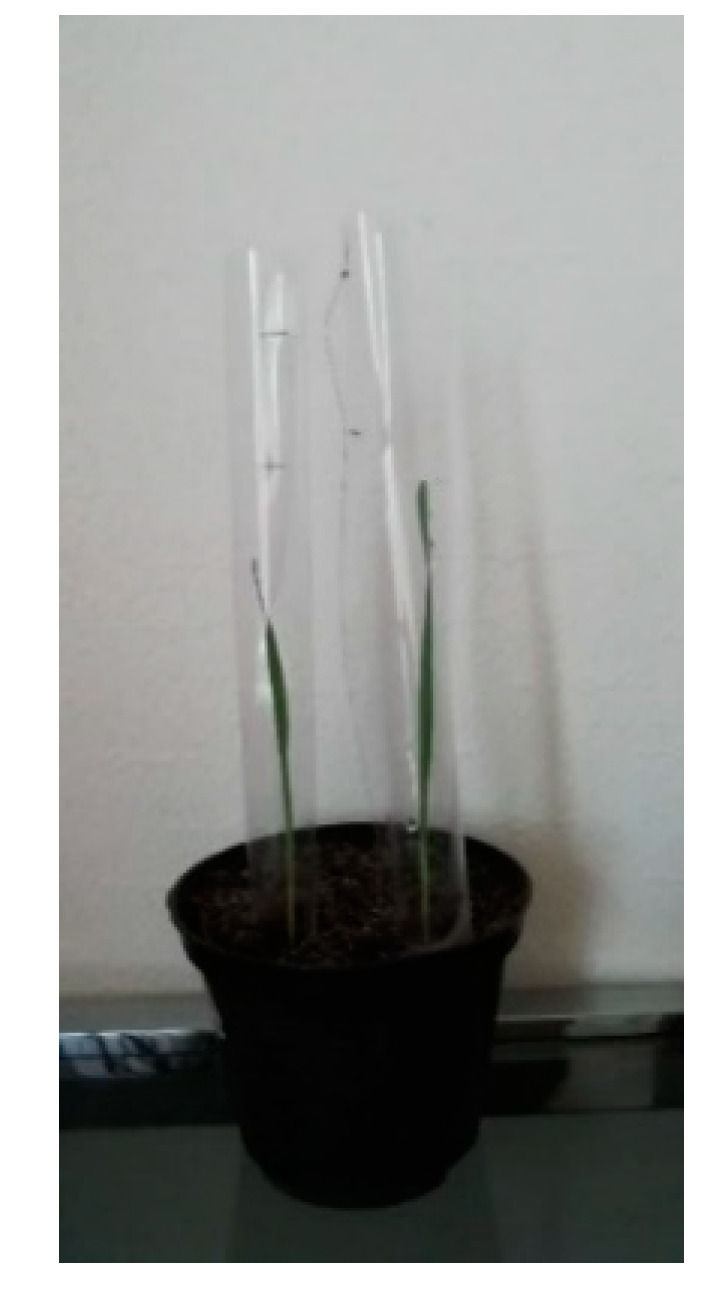
Wheat plants in their cylindrical supports to ensure erect growth.

When the basal leaves were 5 to 6 cm long, single seedlings of Trinakria and Cappelli or Trinakria and its Water-Mutant, with very similar lengths of the basal leaves (10 cm) and roots (11 cm) were selected to be transplanted together into the individual pots (diameter, 10 cm). The pots were filled with 400 g soil and sand mix (50:50; *v*/*v*) with maximum water-holding capacity of 0.32 g H_2_O/g dry weight, with the bottom lined up with the filter paper (Whatman 3MM). The soil was clay-loam (Typic Chromoxerert) with the following physical and chemical characteristics: 36.9% clay, 50.5% silt, 12.5% sand, 15 mg/kg organic matter, pH 8. After sowing, 120 mL deionized water was added to each pot, and they were placed in the growth chamber with the same photoperiod and temperature cycle, as used during germination. To ensure that all plants grow erectly up, they were embedded in transparent cylinders fixed in the ground (Figure 8). Five days later, when the seedlings were at phase 12–13 of the Zadocks scale [34], the plants were taken to the laboratory for phenotyping.

### 4.2. Dehydration Stress Imposition

**Figure 9:**
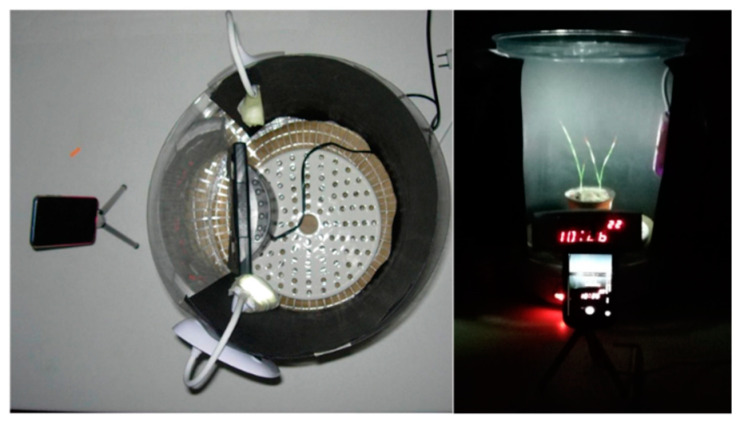
Top (**left**) and front (**right**) views of the instrumentation assembled for the phenotyping of the wilting of the narrow and thin leaves of wheat plants.

The thermostatic heater (FD1065; DCG Eltronics, Brugherio, Italy) used here, was laterally delimited with semi-rigid plastic foil and closed with a lid (Figure 9); this served as the plant incubation chamber and as the ‘movie set’. A ceramic disk, with holes was positioned as the base of the drier to reduce plant vibration due to the movement of the fan, and to better diffuse the heat. To increase the contrast, the back side of the cylinder was covered with a sheet of black paper. The transparent cylinder front was 27 cm long. Two LED lamps were placed on the left and right of the ‘movie set’. The lamps provided about 25,000 lux at plant level. A digital clock with a thermometer was inserted in the camera, to monitor the temperature changes with time through each experiment. In preliminary experiments, the best thermostat setting was identified to have the gradual temperature increase, useful to observe reversible wilting phenomena.

### 4.3. Wilting Experiments

Preliminary wilting experiments were performed to identify the best time–temperature binomial that allowed the recording of both wilting and recovery of the plants or of excised leaves. For each time–temperature binomial, at least three replicate experiments were carried out using the two pairs of genotypes (Trinakria and Cappelli, or Trinakria and its Water-Mutant).

Wilting on plants grown in soil was started when the soil moisture contents ranged from 0.05 to 0.08 g H_2_O/g dry weight. The soil moisture content was determined gravimetrically, because the dry weight of each pot was determined at sowing. By fixing the maximum temperature of the incubation chamber to the value of 48 °C, the optimal duration of stress was ~2.5 h.

The wilting of plantlets obtained from seeds germinated in the rolled paper with single leaves (~10 cm long) was also analyzed, taking the maximum temperature of the incubation chamber to 32 °C. Two plantlets per genotype were inserted into the holes of a rectangular polyurethane sponge, and fixed using the same polyurethane fragments obtained from cutting the holes in the sponge. The roots were blotted dry and protruded down into an empty plastic pot (10 × 10 × 2 cm^3^). Recovery after ~45 min was monitored by turning off the dryer and by adding water into the sponge container.

To analyze the wilting of excised leaves, the second (basal) leaves of six plants of each genotype were cut underwater from well-watered plants, when the soil was at maximum water-holding capacity (0.32 g H_2_O/g dry weight). After cutting, the leaves were immediately inserted into the holes of the polyurethane sponge that floated on the surface of deionized water. The cut ends of the leaves protruded from the bottom, to remain immersed in water. As comparisons of one or two leaves per pair of genotype, at least three wilting experiments were performed. The maximum temperatures ranged from 40 to 45 °C in each experiment. Stress duration ranged from a minimum of 20 min to ~1 h. Recovery was monitored by turning off the dryer.

Simultaneous wilting of four adult leaves per genotype was also analyzed, using fully expanded flag leaves collected from field-grown plants. In the laboratory, the apical sections of the leaves were cut under water to avoid cavitation, and brought to full turgor by immersion for 2 h under distilled water. After the surfaces of the leaves were blotted dry, they were inserted vertically into the holes in the polyurethane sponge, and put in the drier to record the wilting process. In this case, the cut ends of the leaves were not immersed in water. The maximum temperature used was 48 °C, and this was applied for ~45 min. The recovery phase was not recorded.

### 4.4. Camera and Imaging Set-Up

The leaves of the wheat seedlings, plantlets, and single leaves were seen to bend downward when progressing dehydration stress, and for this reason the dynamic analysis of the leaf-tip movement was filmed, according to the measures of the changes in the leaf angle relative to the pot surface. The imaging system used a smartphone equipped with an eight megapixel fully high-definition video recording camera, with a resolution of 1920 × 1080 pixels, which was mounted on a tripod positioned in front of the thermostatic chamber (Figure 9). The free Android App ‘Time-lapse Video Editor’ was installed on the smartphone. The software parameters for the filming were: frame interval, 4 s; video duration, 3 min; speed, 120×; and recording duration, 6 h. The camera was in autofocus mode and the filming was done in a dark room. To improve the tracking of the leaf movements, the tips of the leaves were marked with 4 × 8 mm^2^ orange or red pieces of ‘scotch tape’, the weight of which (0.002 g) ranged from 1% to 3% of the leaf weight. At the end, the movie created was saved with the “.avi” extension for subsequent leaf motion analyses by the Kinovea software.

### 4.5. Motion Analysis

The computerized image processing was performed using the Kinovea 8.25 software, from Kinovea.org, as provided by the University of British Columbia, Canada. Using the commands at the base of the Kinovea video screen, the angles were traced for each of the leaves of the plants, whereby one side coincided with the pot surface, the vertex coincided with the plant collar, and the end of the other side of the corner was fixed on the leaf tip. Using the ‘hand’ symbol and clicking on the corner, the active angle was named before starting the angle movement tracking. At the end of the movie processing, the third icon (film) on the right at the bottom of the video screen was used to save the movie, in ‘.avi’ format. Leaf angle changes with movie time were saved as Excel files. Velocities of folding and unfolding were calculated for each leaf.

To compare the results obtained in replicate experiments that differed for the duration of the wilting process, the velocity of leaf folding was expressed as relative values of leaf angle variation with time, normalized to the maximum movie duration of each experiment. The t-test was used for comparisons of mean values.

Interested readers who wish to see a representative movie will find one in the Supplementary Materials S1 and S2 available online.

## Figures and Tables

**Table 1:** Parameter means obtained by the Kinovea elaboration of the leaf angle changes with time during four wilting experiments on the wild type (WT) Trinakria and Water-Mutant (WM) plants. Mean values (*n* = 8) and significance of genotype comparison by the paired *t* tests are shown.

Statistic	Start of Folding (Rel. Units)		Maximum Angle Change (%)		Velocity of Folding (Rel. Units)		Velocity of Unfolding (Rel. Units)		Recovered Leaf Angle (%)	
	WT	WM	WT	WM	WT	WM	WT	WM	WT	WM
Mean	184.4	173.0	−127.4	−24.6	0.63	0.15	1.38	1.70	−84.7	−8.7
Std error	81.7	79.7	22.6	10.1	0.26	0.10	0.54	0.79	28.7	2.4
*t* test	0.561		4.151		2.163		2.392		2.987	
*P* level	0.5835		0.001 ***		0.0483 *		0.036 *		0.01 **	

*, **, *** Significantly different from zero at 0.05, 0.01, 0.001 probability levels, respectively.

## Supplementary Materials

The following are available online at https://www.mdpi.com/2223-7747/9/6/718/s1, Supplemental Video S1: Video showing the genotypic comparison for the wilting of Trinakria and Cappelli plants grown in soil, for their angle changes with time, from the Kinovea software analysis of the leaf motion in the movies. Supplemental Video S2: Video showing the genotypic comparison for the wilting of excised flag leaves of four leaves of Trinakria (on the left) and four leaves of Cappelli (on the right) and their angle changes with time, from the Kinovea software analysis of the leaf motion in the movies.
